# Marital Choices in the 19th-Century Poznań: Interplay of Origin, Occupation, and Age at Marriage

**DOI:** 10.1007/s12110-026-09511-6

**Published:** 2026-02-10

**Authors:** Linda Koníková, Grażyna Liczbińska, Miroslav Králík

**Affiliations:** 1 Department of Anthropology, Faculty of Science, Masaryk University, Kotlářská 2, Brno, 61137 Czech Republic; 2 The Institute of Human Biology and Evolution, Faculty of Biology, Adam Mickiewicz University, Poznań, Poland

**Keywords:** Marriage, Partner choice, Marital age, Reproductive potential, Socioeconomic status, Place of origin

## Abstract

The study of partner preferences in historical populations is constrained by the limited information available in historical sources. These limitations make it difficult to assess the role of characteristics such as physical attractiveness or psychological traits in spouse selection. Instead, this study examines patterns of marriage partner choice among 19th‑century residents of Poznań, Poland, with particular attention to observable factors including place of origin, occupation, denomination, and age at marriage. The research material contained individual information on 15,652 marriages contracted between 1830 and 1900 in seven Poznań parishes. The partners were divided into four categories based on their marital status: first marriages, marriages of remarried men or women, and second marriages. The analyses included correlation, linear regression, Analysis of Variance, and chi-square tests. First and second marriages differed in the age at marriage of men and women, suggesting that motivations and criteria for marriage changed with life stages and marital history. Specifically, individuals entering the first marriages were the youngest, while divorced or widowed individuals who married within their own marriage category were older than those who married someone who had never been married. First marriage individuals had smaller age differences, but the lowest age correlations compared to all other categories, except remarried women who often married men of similar age or even younger. Across all marriage categories, partners exhibited strong homogamy in denominational, geographical, and social backgrounds, emphasizing the influence of social and cultural factors on marital choices. The origin of the spouses played an important role in partner selection, revealing the impact of the geographical context on the timing of marriage. Specifically, marriages were delayed when at least one partner originated from an urban area and in migrants compared to local population. Marital decisions were also shaped by age and social position, e.g., older men with higher socioeconomic status were more likely to marry younger women. However, these partner selection criteria differed between first and subsequent marriages. The findings of our study support established concepts of marriage patterns while illustrating how biological considerations, geographical contexts, social norms, and economic forces jointly shaped personal choices in historical societies.

**Supplementary Information:**

The online version contains supplementary material available at 10.1007/s12110-026-09511-6.

## Introduction

Marriage patterns provide crucial insights into the social, economic, and cultural background of past societies. Decisions about whom and when to marry were shaped by individual preferences, such as the desire for a similar age, education, or social status, and by structural constraints, including inheritance strategies, land transfers, and migration opportunities (Bras & Kok, 2005; Bull, 2005; Dribe & Lundh, 2005; Van Leeuwen & Maas, 2002). These marital decisions influenced the distribution of resources and reproductive success across generations and revealed the persistence of social norms and the adaptive strategies individuals employed in response to changing environments.

Research shows that age, place of origin, occupation, and socioeconomic status are the important criteria for partner selection (e.g., Dribe & Lundh, 2010; Lippényi et al., 2017; Pollet & Nettle, 2007; Tsou et al., 2011; Van De Putte & Matthijs, 2001). These individual preferences are shaped by, and reflect broader societal changes, such as urbanization, industrialization, and migration, which alter the available marriage market and influence marital choices (e.g., Clark & Cummins, 2022; Foreman-Peck & Zhou, 2018; Lippényi et al., 2017; Moreels & Matthijs, 2011). Although homogamy in demographic and socioeconomic traits predominates – spouses often match in age, education, denomination, or political orientation (e.g., Buss, 2007; Eckland, 1982; George et al., 2015; Hur, 2003; Watson et al., 2004; Zietsch et al., 2011), heterogamy often emerges in terms of women’s age at marriage and men’s socioeconomic status (SES). According to the evolutionary explanations of partner selection, women usually prefer partners with higher social status and resources, while men, especially those of higher SES, prefer younger women (e.g., Anderson & Klofstad, 2012; Barrett et al., 2001; Buss, 1989; Buunk et al., 2002; Hatfield & Rapson, 1996; Kenrick & Keefe, 1992; Pawlowski & Dunbar, 1999). These differing preferences have been explained through evolutionary pressures arising from distinct reproductive roles, each aimed at maximizing reproductive success. For women, marrying a man of higher SES is associated with securing resources for offspring, whereas female youth is often valued as an indicator of health and fertility (Barrett et al., 2001; Buss, 2007; Geary et al., 2004; Pollet & Nettle, 2007; Tsou et al., 2011).

In studying mate choice, it is important to distinguish between homogamy shaped by social constraints and homogamy driven by active preferences. Social homogamy emerges from structural factors such as shared social environments, cultural backgrounds, or geographic proximity that limit the pool of available partners. In contrast, homogamy as a result of active mate choice reflects individual preferences and deliberate selection of partners who resemble themselves in specific traits (Buss, 1985; Luo, 2017; Nagoshi & Johnson, 1994; Watson et al., 2004). These distinctions become particularly salient when considering geographical origin and migration. Strong homogamy, shaped by social and geographic proximity, was observed in rural areas where options for meeting diverse partners were limited (Dribe & Lundh, 2005). Urban residents, by contrast, faced a more diverse marriage market, providing greater opportunities for active mate choice. As a result, they exhibited greater social and cultural heterogamy, married later, and often remained single due to socioeconomic factors and the composition of urban populations. Later marriages were also common among migrants (Lynch, 1991; Moreels & Matthijs, 2011).

Age at marriage has emerged as a particularly important demographic indicator, with John Hajnal’s work identifying a distinctive “European Marriage Pattern” characterized by relatively late marriage and high proportions never marrying in Western and Northern Europe (Hajnal, 1965). In contrast, Eastern European populations typically exhibited earlier and more universal marriage patterns. Evidence from Polish urban parishes demonstrated regional variation, with marriage ages falling between Eastern and Western European norms, reflecting the complex demographic dynamics that likely influenced mate selection (Guzowski, 2013). Similarly, Szołtysek’s (2007) study of the Upper Silesian parish of Bujaków revealed a hybrid age-at-marriage pattern, challenging overly rigid geographic models of family systems.

Despite extensive research on partner selection in Western Europe, much less is known about these dynamics in historical Polish populations. To address this gap, this study draws on newly digitized 19th‑century marriage records from Poznań, a dynamic city under Prussian administration and an important crossroads of migration routes. As the capital of the Poznań province within the partitioned territories, the city experienced intense social, political, and demographic transformations during this period (Liczbińska, 2015). Its demographic composition was ethnically and religiously heterogeneous, with significant populations of Poles, Germans, and Jews, alongside pronounced social and economic stratification. Over the 19th century, Poznań experienced substantial economic growth, urban expansion, and waves of migration, all of which contributed to complex social processes.

Factors such as religious diversity, migration, and occupational structure likely shaped patterns of homogamy and heterogamy in urban Poland. Yet despite their importance for understanding how social and economic capital was transmitted, historical patterns of mate selection in Polish cities remain little studied. By analyzing these factors, this study highlights the interplay between family strategies and social structure, addressing a major empirical gap in the Polish case and contributing to comparative debates on both universal and context‑specific dynamics of mate selection.

### Main Aims

Understanding mate selection in 19th-century Poznań presents interesting yet underexplored research opportunity. Historical studies of mate preferences face inherent challenges, as available sources rarely include information on partners’ biological or psychological traits. Typically, they document only cultural and socioeconomic attributes (occupation, social status, residence, religion, occasional income) and age at marriage. To the best of our knowledge, no in-depth study has examined how Polish historical populations chose marriage partners. In addressing this gap this study investigates whether partner selection in 19th-century Poznań aligned with contemporary evolutionary and social theories of partner choice or reflected distinctive local dynamics. Specifically, we analyze how age, social status, geographic origin, and denomination affected marital outcomes, and compare patterns between first and second marriages. Beyond describing historical patterns, our research aims to initiate a broader interdisciplinary discussion on mate selection and expand knowledge about the factors that operate at the stage of choosing a marriage partner and prospective parent. Such choices, particularly among socially disadvantaged groups, may have played a critical role in the emergence and persistence of social and economic inequalities transmitted across generations.

### Research Hypotheses

The following research hypotheses were tested in this work:

1. In first marriages, men were generally older than their wives, with strong correlations between partners’ ages. However, older men in higher‑status occupations were likely to marry younger women, consistent with evolutionary expectations that men’s status reflected their ability to provide resources and stability, while younger women were associated with greater reproductive potential. In contrast, in second marriages, age gaps tended to widen and age correlations weakened, suggesting a shift toward non‑reproductive criteria such as caregiving or economic support.
2. In both first and second marriages, individuals were more likely to choose partners from their own social milieu (same denomination and place of origin). Spousal homogamy was likely reinforced by familial and societal expectations but may have had different implications by sex: for women, it could provide stronger kin support and facilitate cooperative child‑rearing, whereas for men, it likely reinforced social status and lineage continuity.
3. Age at marriage varied with social and environmental conditions: denomination, place of origin (migration status), and type of location (urban or rural area). Women from conservative denominations, rural areas, or local backgrounds tended to marry younger, consistent with reproductive timing and traditional gender roles. For men, marriage age was more closely tied to occupational prospects and economic pressures. Later marriage was expected among both sexes in migrant and urban populations, reflecting social and economic constraints that delayed family formation.

## Materials and Methods

This study utilizes a comprehensive dataset – “The Poznań Historical Population Database” (https://poznandatabase.pl/), which encompasses individual information on Poznań residents, derived from the 19th-century parish records located in the city of Poznań, Poland. The Poznań society during this period under study varied in terms of denomination, social and economic strata, and underwent intensive political, economic and social changes. The city was profoundly affected by the geopolitical changes associated with its annexation by the Prussian authorities, becoming part of the Prussian state. According to the decision of the Congress in Vienna in February 1815, Poznań became the capital of the Grand Duchy of Poznań, which was a part of the Prussian state in both territorial and administrative terms. In the 1840s, the city became the capital of the Poznań Province (*Provinz Posen*), an administrative unit of the Prussian Empire (Liczbińska, 2015).

The database was established based on information collected from three types of parish records: birth, marriage, and death registers, covering seven Poznań parishes. Five of these parishes were Catholic: St. Mary Magdalene, St. Margaret, St. Martin, St. Adalbert, and St. John, while two were Protestant: the Holy Cross and St. Paulus. The data, derived from the 19th-century sources, are publicly available in the Poznań State Archives and thus do not require ethical approval. For the purpose of this work, individual information on 15,652 marriages contracted in Poznań between 1830 and 1900 was processed, following the Recitals 27, 158, and 150, as well as the Article 89 of the Regulation 2016/679 (GDPR), which establishes data protection rules that do not regulate the processing of personal data of deceased individuals. The dataset includes information on spouses’ age at marriage, marital status, denomination, occupation, and place of origin, providing a detailed portrait of marriage patterns from the early 1800s to the turn of the century.

In this study, marriages were divided into four categories based on the marital status of the partners: (1) first marriages, when both partners had never been married before (*N* = 11,313 couples); (2) marriages of remarried men, when a single woman married a divorced or widowed man (*N* = 2,030 couples); (3) marriages of remarried women, when a bachelor married a divorced or widowed woman (*N* = 1,452 couples); and (4) second marriages, when both partners had been married before (*N* = 857 couples). The following variables were included and analyzed in the study: age at marriage of men and women, recorded in full years in the marriage registers; age difference between partners, calculated by subtracting the age at marriage of woman from the age at marriage of man; occupation of men, classified into six categories (1-workers/peasants; 2-craftsmen; 3-servants/service workers; 4-white-collar workers; 5-traders/good owners; and 6-others); denomination of men and women (1-Catholic; 2-Protestant; 3-other denominations, e.g., Jewish, Orthodox); place of origin of men and women (0-originally from outside Poznań, i.e., migrants; 1-originally from Poznań); and type of location of men and women (0-rural; 1-urban). Information on women’s occupations was rarely recorded. In our database, only 4.3% of women had an occupation assigned to them. They were either servants or workers. Additionally, two groups were distinguished across marriage categories: the reproductive group, in which women married under the age of 45, and the post-reproductive group, in which women married at 45 or older.

Statistical methods, including correlation analysis (Pearson correlation coefficient), paired t-test, linear regression, Analysis of Variance (ANOVA), and chi-squared test, were used to examine relationships between variables. One-way ANOVA was performed to assess differences in ages at marriage of men and women and their age differences across marriage categories and reproductive groups. Post-hoc pairwise comparisons were conducted using Tukey’s Honest Significant Difference (Tukey HSD) test. To explore the relationships between age at marriage and various predictor variables, multiple linear regression models were applied. In the first model, the dependent variable was men’s age at marriage, with independent variables including the woman’s age at marriage, the man’s occupation, denomination, origin, and type of location, as well as the woman’s denomination, origin, and type of location. In the second model, the dependent variable was women’s age at marriage, with independent variables including the man’s age at marriage, occupation, denomination, origin, and type of location, as well as the woman’s denomination, origin, and type of location. The linear regression models were fitted separately for each marriage category. ANOVA was performed on the regression models to assess the overall effects of predictors using the Anova() function from the car R package (Fox & Weisberg, 2019). Type II sum of squares was used to evaluate the effect of each predictor while accounting for the main effects of all other variables in the model. Plots were generated using the ggplot2 R package (Wickham, 2016).

## Results

### Age at Marriage of Men and Women

**Fig. 1:**
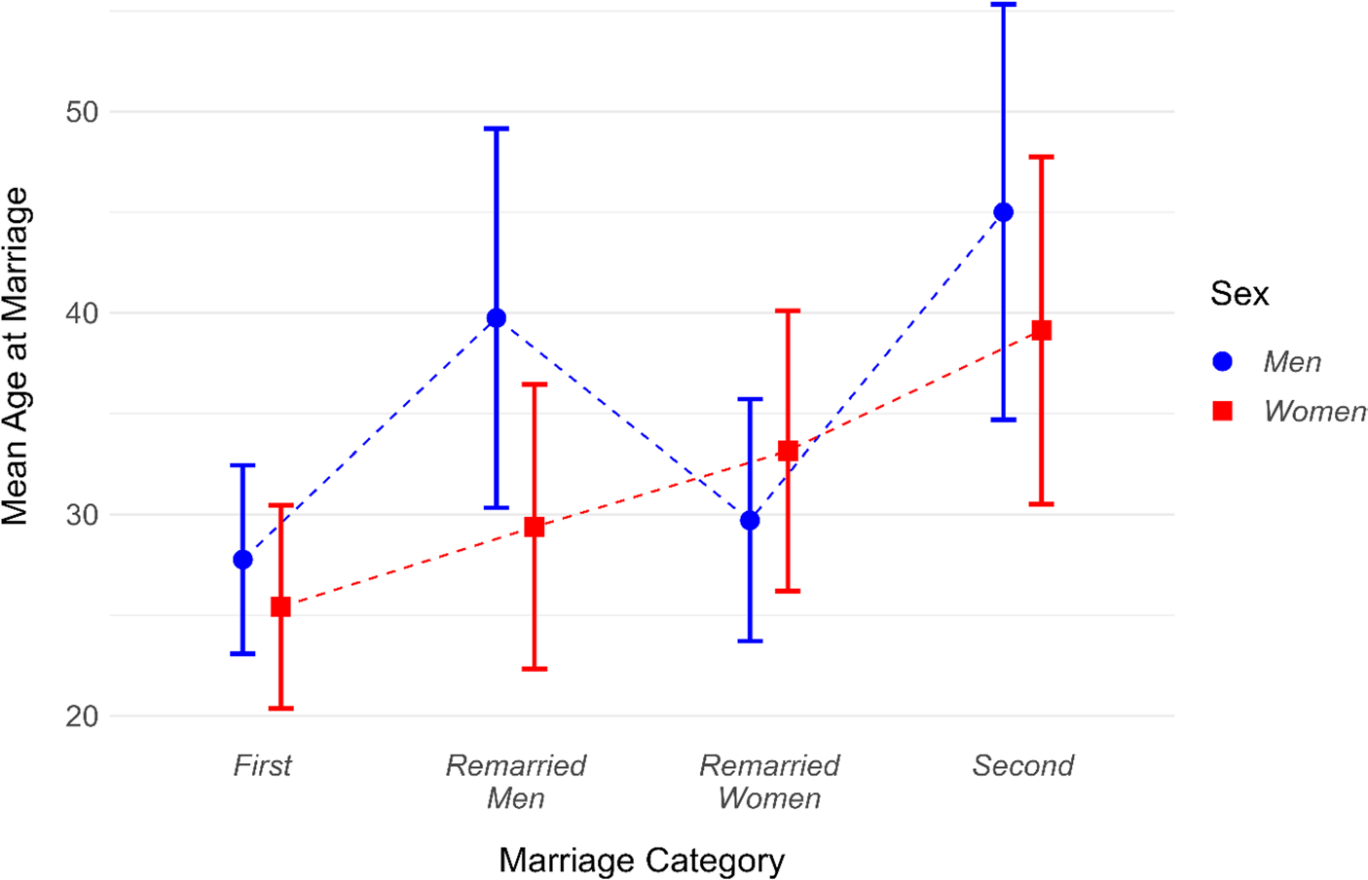
Mean ages at marriage with standard deviation for men and women by marriage category. Significant differences were found between spouses within each category and between all category pairs (*p* < 0.001)

Figure 1 shows mean ages at marriage for men and women across four marriage categories, with line plots illustrating means and standard deviations for each group. Descriptive statistics and correlations between partners' ages are presented in Table 1.

#### Between-Category Variation

One-way ANOVA revealed significant variation in marriage age across categories for both men (F = 3978, *p* < 0.001) and women (F = 2164, *p* < 0.001). Post-hoc comparisons (Table 2) showed that individuals in second marriages were consistently the oldest. On average, men in second marriages were 17.2 years older than men in first marriages, while women in second marriages were 13.7 years older than women in first marriages.

#### Within-Couple Variation

Paired t-tests revealed significant age differences between spouses in all marriage categories (for all four tests *p* < 0.001). Men were older than women in three categories: by 2.4 years in first marriages (t(11235) = 43.29), 5.9 years in second marriages (t(848) = 18.30), and 10.4 years in remarried men marriages (t(2013) = 52.74). Only in remarried women marriages were women older than their partners, by an average of 3.4 years (t(1437) = − 17.39).

#### Partner Age Correlations

Spousal ages were significantly correlated across all categories (*p* < 0.001), ranging from *r* = 0.30 in first marriages to *r* = 0.53 in second marriages (Table 1).

**Table 1:** Mean age at marriage of men (M) and women (F), and spousal age differences (Diff) by marriage category. Values show means with standard deviations (Sd), ranges (Min–Max), and correlations (r) between spouses’ ages within each category

Category	*N*	Group	Min	Mean	Sd	Max	*r*
First marriages	11313	M	16	27.76	4.682	62	0.300
		F	15	25.40	5.047	67	
		Diff	-38	2.35	5.763	35	
Remarried men	2030	M	20	39.74	9.410	78	0.457
		F	16	29.38	7.061	64	
		Diff	-21	10.36	8.818	56	
Remarried women	1452	M	19	29.71	6.001	68	0.340
		F	15	33.14	6.949	70	
		Diff	-41	-3.43	7.484	33	
Second marriages	857	M	18	45.00	10.327	80	0.528
		F	18	39.13	8.616	67	
		Diff	-28	5.85	9.312	37	

**Table 2:** Pairwise comparisons of age at marriage and spousal age differences across marriage categories. Values show mean differences between category pairs with 95% confidence intervals (all comparisons *p* < 0.001)

Comparison	Age differences between women	Age differences between men	Age difference between partners
Second marriages vs. First marriages	13.72 [13.20, 14.25]	17.22 [16.66, 17.77]	3.49 [2.89, 4. 10]
Second marriages vs. Remarried women	5.99 [5.34, 6.63]	15.26 [14.59, 15.94]	9.28 [8.54, 10.02]
Second marriages vs. Remarried men	9.75 [9.14, 10.36]	5.23 [4.59, 5.86]	-4.52 [-5.21, -3.82]
Remarried women vs. First marriages	7.74 [7.32, 8.16]	-1.95 [-2.39, -1.52]	-5.79 [-6.26, -5.31]
Remarried women vs. Remarried men	3.76 [3.25, 4.28]	10.04 [9.50, 10.57]	-13.79 [-14.38, -13.21]
First marriages vs. Remarried men	-3.98 [-4.33, -3.62]	11.99 [11.61, 12.36]	8.01 [7.60, 8.42]

### Age Differences Between Partners

**Fig. 2:**
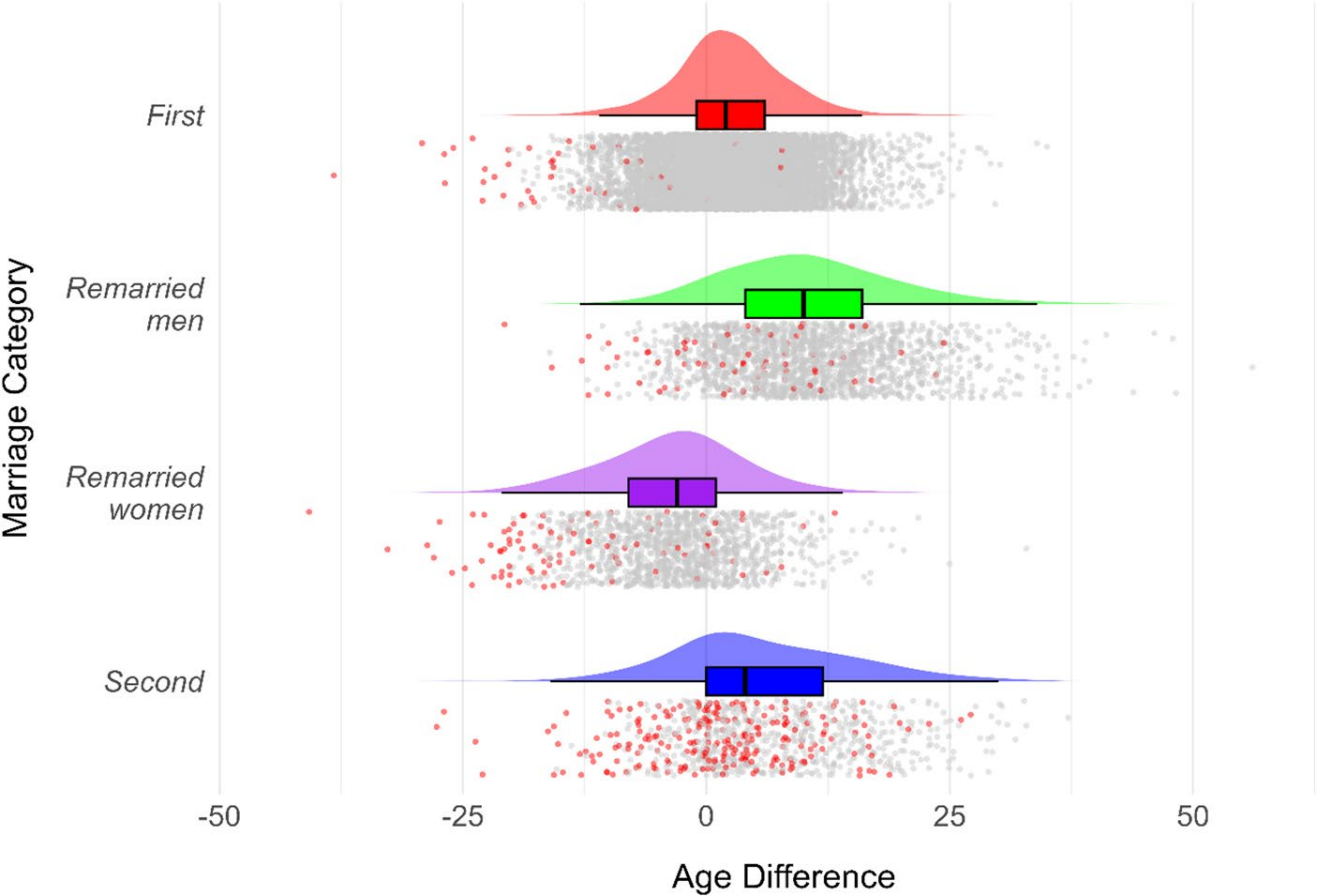
Raincloud plots of spousal age differences by marriage category. Points representing marriages with post-reproductive women (aged 45+) are highlighted in red

**Fig. 3:**
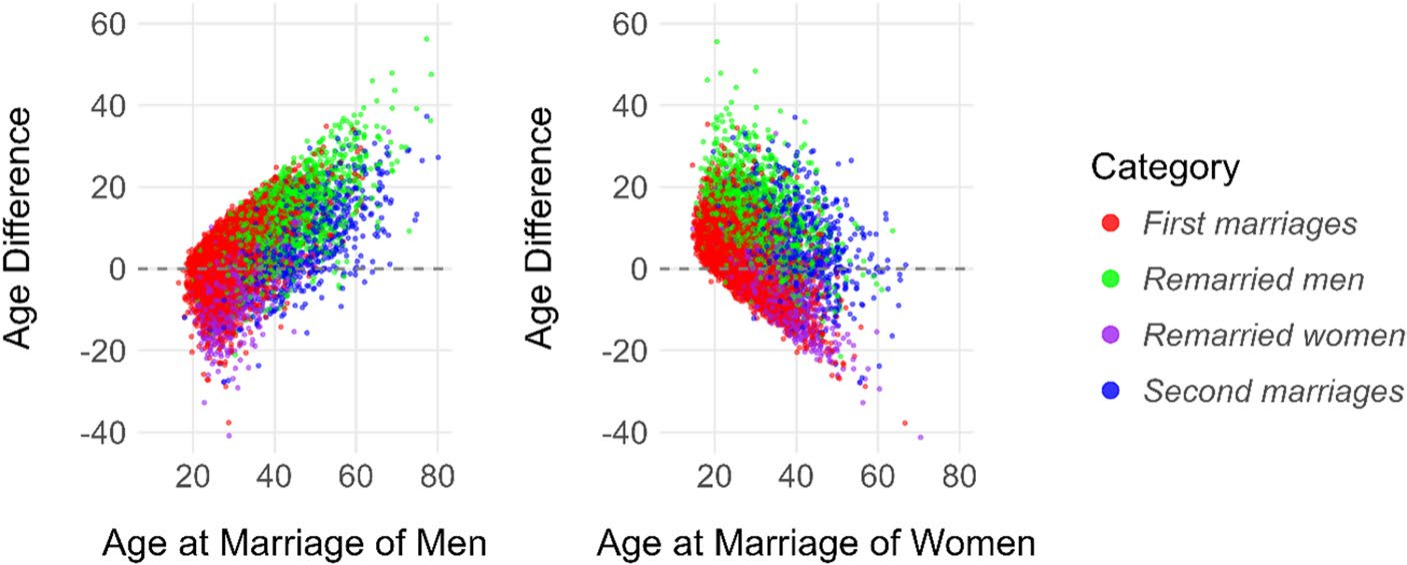
Scatterplots of spousal age differences in relation to men’s age at marriage (left) and women’s age at marriage (right), by marriage category

Figures 2 and 3 illustrate the distribution of age differences and their relationships with partner ages across marriage categories.

#### Between-Category Variation

Age differences between partners varied systematically across marriage categories (Table 1). Remarried men showed the largest age differences, followed by second marriages and first marriages. Remarried women marriages showed the negative age difference between partners. ANOVA confirmed significant differences in age differences between categories (F = 1365, *p* < 0.001), with post-hoc comparisons (Table 2) showing significant differences between all category pairs (*p* < 0.001).

#### Correlations of Age Difference

Correlation analyses (Table 3) revealed that age differences were positively associated with men’s age and negatively associated with women’s age across all categories (*p* < 0.001). This suggests that older grooms tend to have larger age differences with their partners, while older brides tend to have smaller age differences.

**Table 3:** Pearson correlation coefficients between spousal age differences and age at marriage of men and women, by marriage category

Category	Correlation with age at marriage of men (*r*)	Correlation with age at marriage of women (*r*)
First marriages	0.550	-0.632
Second marriages	0.618	-0.341
Remarried men	0.701	-0.314
Remarried women	0.487	-0.656

### Reproductive Status of Women

The proportion of marriages involving women over 45 years (post-reproductive age) varied significantly by marriage category. Second marriages had the highest proportion (27.6%, *N* = 234), followed by remarried women marriages (6.5%, *N* = 93), remarried men marriages (3.6%, *N* = 72), and first marriages (0.5%, *N* = 53). ANOVA revealed significant differences between reproductive groups in both men’s marriage age and age differences between partners (*p* < 0.001). Men marrying post-reproductive women were 16.6 years older on average than men marrying reproductive-age women. Additionally, age differences between partners were 5.8 years larger in the reproductive group compared to the post-reproductive group. Figure 2 shows the distribution of age differences by reproductive status.

### Milieu Homogamy

The tendency to marry within the same social milieu was assessed for denomination, place of origin, and urban versus rural location using chi-squared tests of independence. Separate contingency tables were constructed for each milieu across the four marriage categories (Table 4), yielding twelve tests in total, all of which were significant (χ² *p* < 0.001). In every marriage category, same-denomination unions outnumbered inter-denominational marriages. Similarly, spouses overwhelmingly shared the same place of origin, most commonly both Poznań natives, with migrant men marrying women from Poznań as the second most frequent. Finally, urban–urban and rural–rural marriages were more common than marriages with mixed location types.

**Table 4:** Counts (with percentages) of male–female pairings by denomination, place of origin, and type of location across four marriage categories. Each milieu was tested separately, resulting in twelve contingency tables, all of which were significant (χ² *p* < 0.001)

Milieu	Combination	First marriages	Second marriages	Remarried men	Remarried women
Denomination	Catholic-Catholic	7 435 (65.80%)	712 (83.08%)	1 502 (74.03%)	1 119 (77.39%)
	Protestant-Protestant	2 898 (25.65%)	107 (12.49%)	447 (22.03%)	265 (18.33%)
	Catholic-Protestant	323 (2.86%)	10 (1.17%)	18 (0.98%)	17 (1.18%)
	Protestant- Catholic	605 (5.35%)	25 (2.92%)	59 (2.91%)	42 (2.90%)
	Other	38 (0.34%)	3 (0.35%)	3 (0.15%)	3 (0.21%)
Place of origin	Poznań- Poznań	7 027 (74.38%)	536 (77.01%)	1 323 (77.64%)	908 (77.61%)
	Migrant-Migrant	842 (8.91%)	61 (8.76%)	121 (7.10%)	102 (8.72%)
	Migrant- Poznań	1 322 (13.90%)	87 (12.50%)	204 (11.97%)	138 (11.79%)
	Poznań-Migrant	257 (2.72%)	12 (1.74%)	56 (3.29%)	22 (1.88%)
Type of location	Urban-Urban	6 323 (66.95%)	450 (64.66%)	1 147 (67.31%)	733 (62.65%)
	Rural-Rural	1 843 (19.51%)	179 (25.72%)	324 (19.01%)	307 (26.24%)
	Rural- Urban	822 (8.70%)	46 (6.61%)	154 (9.04%)	76 (6.50%)
	Urban-Rural	457 (4.84%)	21 (3.02%)	79 (4.64%)	54 (4.62%)

### Social and Demographic Predictors of Age at Marriage

The results of the multiple linear regression are presented in Table S1. The linear models were fitted separately for each marriage category to examine the relationship between the age at marriage of men and women and specific category effects. The relatively low R-squared values suggest that the age at marriage of partners may have been influenced by additional factors not captured in this study. The results of the ANOVA, assessing the overall importance of the predictors, are presented in Table S2.

#### Age at Marriage

**Fig. 4:**
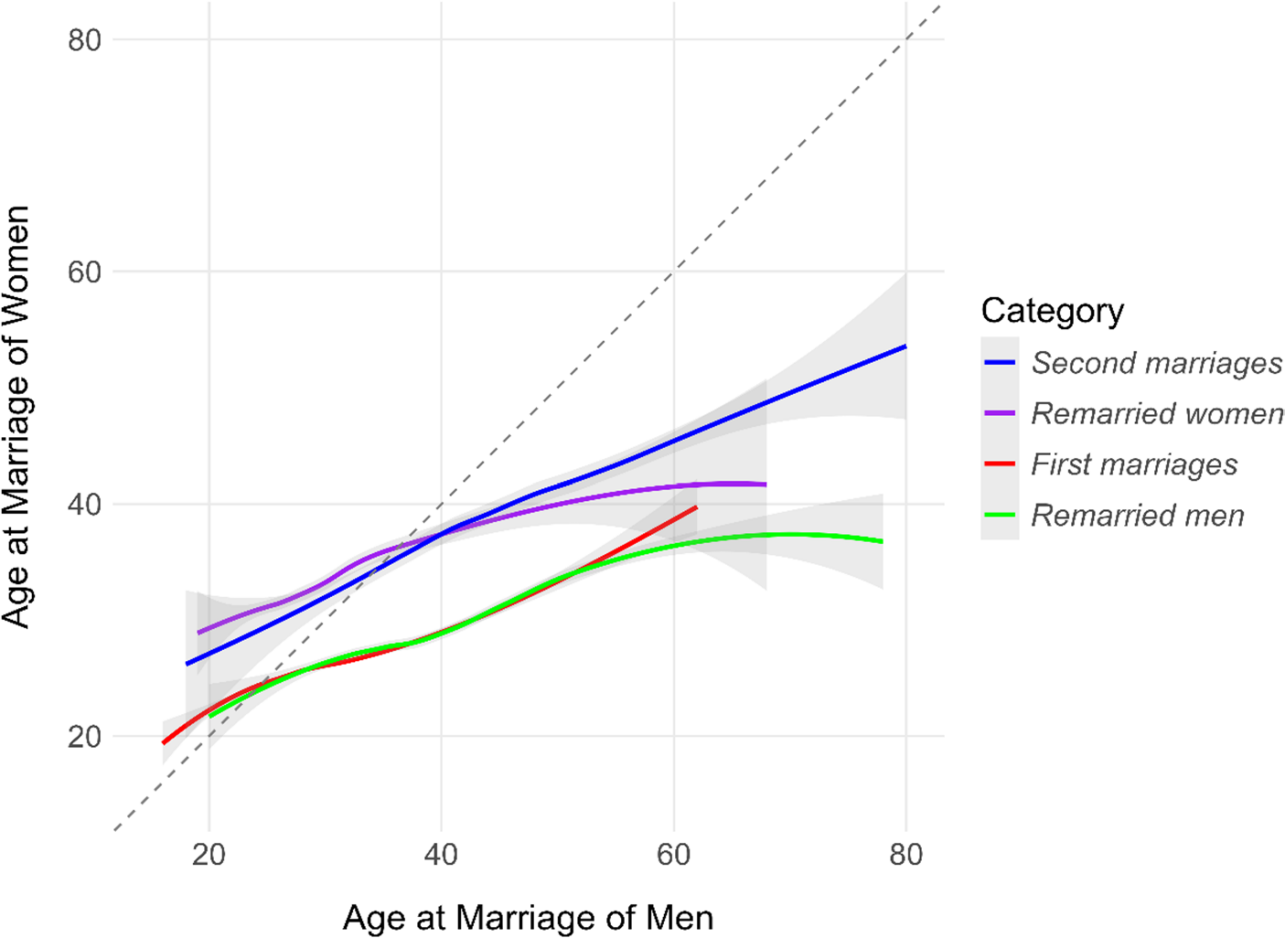
The relationship between the age at marriage of men and women by marriage category. The scatter plot includes a LOESS (Locally Estimated Scatterplot Smoothing) curve, with a shaded area representing the standard error of the mean. The diagonal line represents equal ages for both partners

The regression analysis (Table S1) indicates that older men married older women in all marriage categories (as the age of the groom increases, so does the age of the bride). However, the regression coefficients were relatively low for first marriages and marriages of remarried women (0.283 and 0.279, respectively). This means that, on average, the bride was only about 0.28 years older for every year the groom was older, suggesting that older men tended to marry relatively younger women. The regression coefficients were higher for second marriages (0.620) and marriages of remarried men (0.629) than for first marriages. Based on ANOVA (Table S2), the age at marriage of women was the strongest significant predictor, explaining most of the variance in the age at marriage of men across all marriage categories. Women tended to marry slightly older as the age of their partners increased (for each additional year of age of the groom, the age of the bride increased by about 0.329 years for first marriages, 0.354 years for marriages of remarried men, 0.388 years for marriages of remarried women, and 0.448 years for second marriages). The age at marriage of men was the strongest significant predictor of the age at marriage of women across all marriage categories. The relationship between the age at marriage of partners is shown in Fig. 4.

#### Groom’s Occupation

**Fig. 5:**
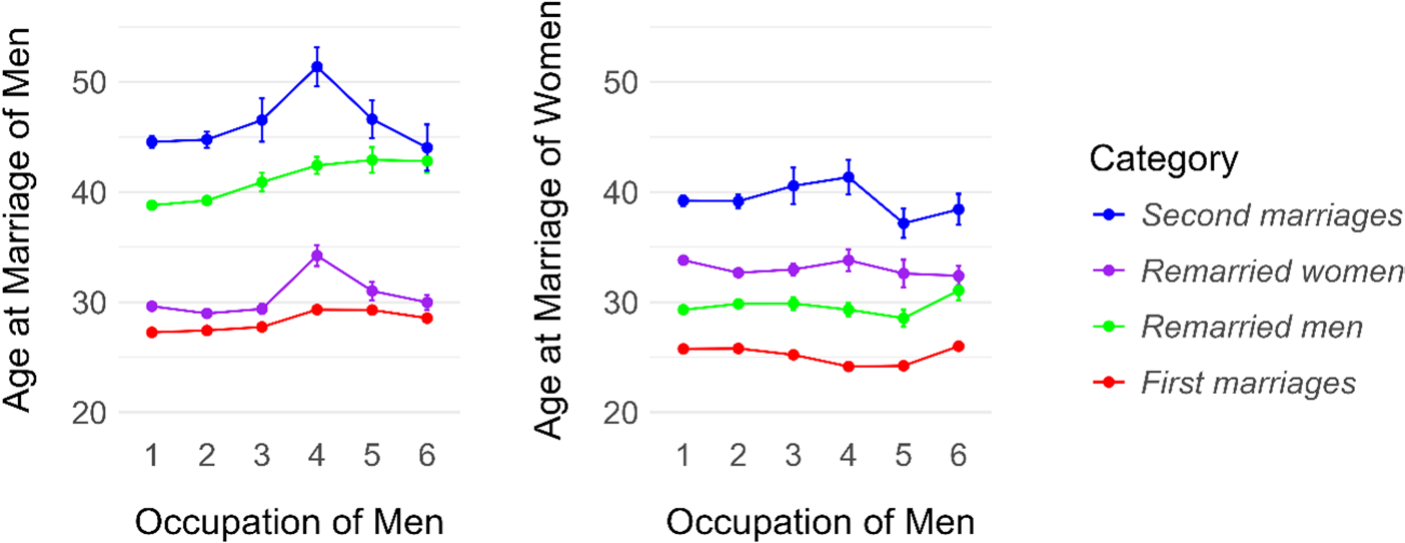
Mean age at marriage of men (left) and women (right) by occupation of men (1-workers/peasants; 2-craftsmen; 3-servants/service workers; 4-white-collar workers; 5-traders/good owners; 6-others) and marriage category. Error bars indicate the standard error of the mean

Table S1 shows that in first marriages, men employed in the service sector tended to marry about 0.541 years older than the baseline occupation (manual workers). White-collar workers married about 2.202 years older, traders/good owners about 2.255 years older, and men in other occupations about 1.019 years older than workers. A similar pattern can be seen for remarried men: white-collar workers, traders/good owners, and men in other occupations married about 3.452 years, 4.615 years, and 2.573 years older than workers, respectively. In second marriages, and among remarried women, white-collar workers tended to marry about 3.957 and 4.231 years older than blue-collar workers. Only craftsmen within the category of remarried women tended to marry about 1.041 years younger than workers. Based on Table S2, the groom’s occupation significantly impacted the age at marriage of men, except for second marriages. Women who married craftsmen were about 0.611 and 0.957 years younger than women who married workers, both in first and remarried men marriages. Women who married servants were about 0.901 or 1.477 years younger, those who married white-collar workers were about 2.617 or 2.304 years younger, and those who married traders/good owners were about 2.451 or 3.677 years younger. In second marriages, women marrying traders/good owners were about 3.034 years younger than those who married workers. Based on ANOVA, the groom’s occupation had a significant effect on the age at marriage of women in the category of first and remarried men marriages, aligning with the linear model results. The relationship between the mean age at marriage of men and women and the occupation of the groom is shown in Fig. 5.

#### Denomination

The denomination of the groom or the bride had no significant effect on the age of the groom or bride at the time of marriage, with the exception of second marriages. For the second marriages, the denomination of men had a significant effect on the age at marriage of women, who were about 3.906 years older when married to Protestant men than when married to Catholic men.

#### Place of Origin

In first marriages, the origin of men had a significant effect on their age at marriage: men from Poznań entered into first marriages about 0.908 years earlier than men from outside Poznań. The origin of the bride had no significant effect on the groom’s age at marriage. Furthermore, in first marriages, the origin of women and their partners had a significant effect on their age at marriage: women from Poznań married about 0.468 years earlier than those from outside Poznań. However, women (of all origins) married about 0.895 years later if their partners were from Poznań.

#### Type of Location

In first marriages, the type of location of men was a significant predictor of their age at marriage: men from urban areas entered into first marriages about 0.741 years older than those from rural ones. Furthermore, the type of location of women was also a significant predictor of the age at marriage of men: in the groups of first, second, and remarried women marriages, men who married urban women were about 0.543, 3.039, and 1.912 years older, respectively, than those who had a spouse from rural areas. In other words, men from urban areas married later than men from rural areas, and men who married women from urban areas also tended to be older. Finally, in first and remarried men marriages, the type of location of women had a significant effect on their age at marriage: women from urban areas married about 1.917 and 1.958 years older than women from rural areas. In addition, the type of location of men had a significant effect on the age at marriage of women in marriages of remarried men: women who married for the first time with remarried men from urban locations were about 1.477 years older than those who entered into matrimony with remarried men from rural locations.

## Discussion

Marriage records are among the most complete and reliable sources of information on past societies. Great significance was attached to them out of concern for the legitimacy of offspring (Liczbińska, 2015). Unlike other life events, which may be underreported or inconsistently documented, marriage records typically provide a comprehensive account, offering valuable insights into the social, economic, and cultural background of historical societies. This study analyzed marriage records to examine factors influencing age at marriage and selection criteria among 19th-century spouses in Poznań.

In the first hypothesis, we assumed that patterns of age at marriage differed between first and second marriages. This is because the criteria for selecting a partner, reasons for getting married, and the social acceptability/approval of marriage may have differed. Our study confirmed this hypothesis. The mean age at marriage of men and women varied across marriage categories, with the youngest individuals entering into first marriages. Regarding other marriage categories, our findings are consistent with earlier observations that individuals often married spouses of a similar age and marital status, while those with atypical ages for their status tended to select partners with matching age but different marital statuses (National Center for Health Statistics & Wilson, 1989): divorced or widowed men who married divorced or widowed women were older than those who married single women. These men may have still sought reproductive opportunities, leading them to choose younger, potentially more fertile partners. Even if they were not as young as bachelors, they could have offered social or economic advantages up to the point at which their increasing age diminished their perceived attractiveness and ability to provide long-term stability in marriage (Conroy-Beam & Buss, 2019). Similarly, divorced or widowed women who married divorced or widowed men were older than those who married bachelors. Younger divorced or widowed women, still in their reproductive years may have sought bachelor partners who had no prior marital obligations. Conversely, these women may have been valued as experienced, emotionally mature, and available, particularly in contexts where previously single women were already married or had other preferences, highlighting the role of marriage market constraints (Conroy-Beam & Buss, 2019). Overall, individuals entering second marriages likely prioritized factors beyond reproduction, as many already had children from prior unions, leading them to choose partners at a similar life stage.

Couples in first marriages tended to be more similar in age. They generally exhibited smaller age differences compared to other marriage categories, a pattern also noted by Buss (1985), with the exception of remarried women, who showed a negative mean age difference. Interestingly, despite this similarity, the correlation between the ages of brides and grooms in first marriages was the lowest. This can be explained by the limited age variance in first marriages, where both partners were typically young. In such cases, being young and close in age was likely sufficient both biologically and socially, making precise age differences less relevant. In contrast, age variance increased in remarriages, resulting in larger age differences and stronger correlations between partners’ ages, with the highest correlation observed among individuals in their second marriages. Furthermore, partner selection in second marriages was likely less complex, given a smaller pool of available partners, e.g., a partner who had lost his or her previous spouse (fewer options) or a partner from a closer area and with similar activities (higher probability of meeting). The reason for marrying may also have been different in second marriages. As both partners are likely to have children from a previous relationship, they may now be looking for companionship and support (psychological and/or financial) in a marriage union. The category of second marriages also included the highest proportion of post-reproductive women. Overall, men who married women of post-reproductive age were older, but age differences in these marriages were smaller than those in unions involving women of reproductive age. Thus, while larger age differences in marriages involving younger women may reflect an emphasis on fertility and childbearing, smaller age differences in marriages with post-reproductive women likely indicate a shift in partner selection criteria – from biological (e.g., having children) to psychological and practical priorities (e.g., companionship and mutual support).

The age differences in second marriages (for both partners) fall between those observed in remarried men and first marriages. While remarried men tended to marry significantly younger brides, leading to the largest age differences compared to all other categories, remarried women showed a distinct pattern. They tended to have smaller age differences, often marrying men of similar age or even younger, a pattern consistently reported by the National Center for Health Statistics and Wilson (1989). This may reflect marriage market dynamics: as divorced or widowed women aged, their mate value declined, making it less likely for them to fulfill their mate preferences, such as seeking an older partner (Conroy-Beam & Buss, 2019). Additionally, it may be related to the differences in life expectancies and remarriage opportunities of men and women. Higher male mortality rates, shorter life spans, and the tendency for women to marry older men, led to more widows than widowers, skewing sex ratios and reducing the pool of available partners for older women (Carr & Bodnar-Deren, 2009; James & Shafer, 2012; Liczbińska, 2015). Moreover, widowed women, particularly those who inherited property from a late husband, could offer financial stability in exchange for companionship to younger men who faced delays in entering the marriage market, perhaps due to military service, economic challenges, or taking care of their younger siblings after their father’s death. Older widows may also have been valued as experienced and capable partners. Finally, marriage norms may have been more flexible for men, allowing them to marry older women without significant stigma, as well as for women in their remarriages, who may have been more accepted in marrying younger partners without much societal disapproval. Although older women marrying younger men can lead to unique relationship dynamics, often influenced by socio-economic factors and shifting societal norms, this area remains largely unexplored (Proulx et al., 2006).

Overall, the age differences depended significantly on the ages of both partners. Older husbands were associated with larger age gaps, indicating a tendency for older men to marry proportionally younger women, whereas older wives were linked to smaller age differences across all marriage categories. Our findings are consistent with those of Bhrolchain (1992), particularly in observing smaller age differences in first marriages compared to second marriages and highlighting sex-based differences in age gaps in remarriages. For divorced or widowed men, the age differences were larger compared to divorced or widowed women who tended to marry partners of similar age (Bhrolchain, 1992).

Our analysis showed that residents of the 19th-century Poznań married partners from similar background in terms of denomination and place of origin. This homogamy applied to both first and second marriages. In the 19th century, mate choice was primarily influenced by religious, social, and physical considerations, as marriage was regarded as a practical institution essential for social order, reinforcing morality, class conformity, and religious values (Gordon & Bernstein, 1970; Van De Putte & Matthijs, 2001). Most marriages occurred within the same social and economic class (Dribe & Lundh, 2010). Regarding homogamy between spouses, we found a strong tendency for marriage within the same denomination group for both first and second marriages. Catholic men primarily married women from the same denomination, and Protestant men and women showed a similar pattern. Mixed-denomination marriages were rare, accounting for a minimal percentage of all marriage types, suggesting a clear tendency toward religious homogamy in these unions, consistent with previous research (e.g., Hur, 2003; Watson et al., 2004; Zietsch et al., 2011).

Regional homogamy was also evident in both first and second marriages. Men from Poznań frequently married women from the same city. However, the second largest group was represented by migrant men marrying women from Poznań, while marriages of spouses both originating from other regions were less common, and men from Poznań marrying migrant women was the rarest combination. This result can be explained by the fact that men predominated among migrants. Moreover, urban men consistently preferred to marry urban women, while rural men predominantly married rural women in both first and second marriages. This result highlights a strong tendency for individuals to choose partners from the same type of location. Overall, confessional and origin homogamy were strongly confirmed, with the notable exception of migrant men marrying women from Poznań, which represented a relatively common deviation from these patterns.

We have partially confirmed that the age at marriage of men and women varied by social and environmental conditions. Denomination generally had no significant effect, likely due to the high prevalence of intra-denominational unions among the majority Christian population. The notable exception was in second marriages, where women married later to Protestant men, potentially reflecting different remarriage norms: Protestant men, often migrants with more permissive attitudes toward widow remarriage, were more likely to marry older women, whereas Catholic remarriages were likely shaped by more traditional concerns.

Regarding the place of origin, migrants (both men and women) entered into their first marriages later than locals. This finding is consistent with broader research on the impact of migration on life course events. Migrants often face delays in marriage due to economic challenges and the need to establish themselves in a new community, i.e., they have to adapt to new environments and work to achieve financial stability before entering into marriage (e.g., Antu et al., 2022; Carlson, 1985; Du, 2023; Lynch, 1991). Interestingly, women (whether migrants or locals) married later when their partner was from Poznań, suggesting that younger women were more likely to marry migrant men.

Urban residence was also associated with delayed marriage for both men and women, particularly in first marriages. Even when only one of the partners was from an urban area, the tendency was to marry later, reflecting that urban environments may have been associated with delayed marriage, possibly due to the need for education, career establishment, and economic stability (and time to migrate) before marriage (Brooks & Clark, 2024; Lynch, 1991; Moreels & Matthijs, 2011; Schmidt et al., 2015). When only one partner originated from an urban area, the tendency to delay marriage may have persisted due to the influence of the urban partner’s social and economic expectations. This pattern may also have reflected the influence of urban social norms on the timing of marriage, as individuals from urban areas may have prioritized stable financial and social conditions over traditional timelines associated with marriage. In contrast, rural areas maintained more traditional family-oriented patterns (Heaton et al., 1989). Single life was less common or less socially acceptable. Moreover, people used to inherit land from their parents; therefore, marriages were strongly influenced by family and community, the need to ensure the continuity of family wealth and property, and to maintain economic and social stability (Bull, 2005; Dribe & Lundh, 2005; Schmidt et al., 2015; Van Leeuwen & Maas, 2002).

Our results confirmed a general tendency toward age homogamy: older men married older women. However, the marriage age of women increased less steeply with that of their partners (Fig. 4), resulting in a widening age gap at higher ages. This finding is consistent with previous research showing men’s stronger preference for younger partners, particularly at later ages (e.g., Buss, 1985, 1989, 2019). The pattern was particularly pronounced in first marriages and marriages of remarried women, reflecting the tendency to marry within similar age groups, likely due to shared social environments and life stages. Nonetheless, despite men typically being older than their wives, there remains a preference for relative age similarity between partners (Van De Putte & Matthijs, 2001).

At the same time, age homogamy is also shaped by socioeconomic class, with wealthier individuals having greater flexibility in choosing partners outside their age range, while lower classes are more constrained by economic and social circumstances (Dribe & Lundh, 2010; Van De Putte & Matthijs, 2001). Regarding the occupation of grooms in relation to the age of their partners, we found that white-collar workers and traders/good owners (representing higher SES strata) tended to marry later than others, likely due to the time required for education and professional experience. Additionally, these men may have remained attractive due to their greater resources and financial stability, while their non-demanding occupations may have helped them to maintain a youthful appearance. This pattern was observed in first marriages and remarried men. For second marriages (including remarried women), the occupation of the groom did not significantly influence age at marriage, except for white-collar workers, who may have needed more time for education, first marriage, and divorce (or to become a widower), and/or who may have been preferred by women for remarriage despite their older age. Furthermore, our results suggest that women married men of higher SES at a younger age. This relationship was no longer significant for second marriages (except for younger women marrying traders/good owners) and for remarried women. Women’s age likely served as a crucial biological indicator, reflecting health, reproductive potential, and fertility (Barrett et al., 2001; Buss, 1989, 2007; Conroy-Beam & Buss, 2019; Geary et al., 2004; Kenrick & Keefe, 1992; Kenrick et al., 1996; Pawlowski & Dunbar, 1999). Moreover, the importance of age preference is heightened in historical populations, when limited access to medical care contrasts with modern societies, where the advancements allow childbirth at older ages. Men’s age, by contrast, was less important than social status and resources, which could be invested in future offspring (Anderson & Klofstad, 2012; Barrett et al., 2001; Buss, 1989, 2007; Buunk et al., 2002; Geary et al., 2004; Kenrick & Keefe, 1992; Pawlowski & Dunbar, 1999; Pollet & Nettle, 2007; Tsou et al., 2011).

Overall, our results suggest that marriages between divorced or widowed men and single women were more similar to first marriages for both partners compared to those of remarried women, highlighting the importance of women’s age in marriage decisions. Men may have prioritized reproductive potential, seeking younger women with whom they could potentially have biological children, replicating the dynamics of the first marriage. Societal norms likely allowed men greater flexibility to remarry younger women, particularly if they brought resources, stability, or social status. In contrast, remarried women’s unions were more likely to reflect second marriage patterns, with partner choice reflecting factors beyond reproduction, such as companionship, support, and life stage compatibility.

## Conclusion

The analysis of more than 15,000 marriages revealed significant variation in both the age at marriage and the relationship between spouses’ ages in first and second marriages, suggesting that marital motivations evolved across life stages. Our results align with evolutionary theories of mate selection by underscoring the role of reproductive potential in marital decisions. At the same time, marital choices were strongly shaped by denominational, geographical, and social homogamy, while migration status, type of location (urban or rural area), and certain occupations of the groom also influenced the timing of marriage. Overall, the study highlights the complex interplay of biological, geographical, social, and economic factors in shaping personal choices in the 19th-century Poznań.

## Supplementary Information

Below is the link to the electronic supplementary material.

Supplementary file 1 (PDF 93.0 KB)

## Data Availability

The data that support the findings of this study are available from the authors upon reasonable request.
