# Supplementary material for: Marital Choices in the 19th-Century Poznań: Interplay of Origin, Occupation, and Age at Marriage
Source: Hum Nat. 2026 Feb 10;36(4):588–608. doi: 10.1007/s12110-026-09511-6 (PMC12946279; doi:10.1007/s12110-026-09511-6)
Supplement: Supplementary file 1 — (PDF 93.0 KB) [file 12110_2026_9511_MOESM1_ESM.pdf]

Table S1 Regression models predicting the age at marriage of men and women (Intercept) by marriage category. Abbreviations: female (F), male (M), occupation (2-craftsmen; 3-servants/service workers; 4-white-collar workers; 5-traders/good owners; 6-other), denomination (2-Protestant; 3-other), origin (1-Poznań), and location (1-urban). Significance codes: 0 '***' 0.001 '**' 0.01 '*' 0.05 '·' 0.1 ' ' 1

	Intercept: Marriage age M	Estimate	SE	t value	p value		Intercept: Marriage age F	Estimate	SE	t value	p value	
First marriages	Intercept	19.687	0.295	66.785	< 2e-16	***	Intercept	15.328	0.356	43.022	< 2e-16	***
	Marriage age F	0.283	0.010	29.193	< 2e-16	***	Marriage age M	0.329	0.011	29.193	< 2e-16	***
	Occupation M2	0.030	0.131	0.227	0.821		Occupation M2	-0.611	0.141	-4.345	0.000	***
	Occupation M3	0.541	0.167	3.231	0.001	**	Occupation M3	-0.901	0.180	-4.994	0.000	***
	Occupation M4	2.202	0.195	11.282	< 2e-16	***	Occupation M4	-2.617	0.210	-12.457	< 2e-16	***
	Occupation M5	2.255	0.219	10.296	< 2e-16	***	Occupation M5	-2.451	0.236	-10.382	< 2e-16	***
	Occupation M6	1.019	0.242	4.215	0.000	***	Occupation M6	-0.286	0.261	-1.094	0.274	
	Denomination M2	-0.008	0.169	-0.046	0.963		Denomination M2	-0.300	0.183	-1.645	0.100	
	Denomination M3	0.580	0.863	0.672	0.501		Denomination M3	-0.580	0.931	-0.624	0.533	
	Origin M1	-0.908	0.157	-5.780	0.000	***	Origin M1	0.895	0.169	5.280	0.000	***
	Location M1	0.741	0.168	4.419	0.000	***	Location M1	0.088	0.181	0.488	0.625	
	Denomination F2	-0.074	0.171	-0.432	0.666		Denomination F2	0.224	0.184	1.217	0.224	
	Denomination F3	0.181	2.001	0.090	0.928		Denomination F3	-1.924	2.158	-0.892	0.373	
	Origin F1	0.198	0.216	0.919	0.358		Origin F1	-0.468	0.232	-2.015	0.044	*
	Location F1	0.543	0.186	2.924	0.003	**	Location F1	1.917	0.199	9.621	< 2e-16	***
Second marriages	Intercept	20.388	2.182	9.344	< 2e-16	***	Intercept	19.038	1.824	10.439	< 2e-16	***
	Marriage age F	0.620	0.043	14.417	< 2e-16	***	Marriage age M	0.448	0.031	14.417	< 2e-16	***
	Occupation M2	-0.239	0.926	-0.258	0.796		Occupation M2	-0.342	0.787	-0.434	0.664	
	Occupation M3	0.545	1.611	0.338	0.735		Occupation M3	0.384	1.369	0.281	0.779	
	Occupation M4	3.957	1.899	2.083	0.038	*	Occupation M4	-1.322	1.620	-0.816	0.415	
	Occupation M5	1.485	1.731	0.858	0.391		Occupation M5	-3.034	1.467	-2.069	0.039	*
	Occupation M6	-1.991	1.960	-1.016	0.310		Occupation M6	-0.971	1.667	-0.583	0.560	
	Denomination M2	-1.097	1.862	-0.589	0.556		Denomination M2	3.906	1.574	2.481	0.013	*
	Denomination M3	10.271	9.057	1.134	0.257		Denomination M3	4.392	7.707	0.570	0.569	
	Origin M1	1.491	1.275	1.169	0.243		Origin M1	-0.559	1.085	-0.516	0.606	
	Location M1	-1.161	1.540	-0.754	0.451		Location M1	0.706	1.310	0.539	0.590	
	Denomination F2	1.983	1.892	1.048	0.295		Denomination F2	-2.757	1.606	-1.717	0.087	.
	Denomination F3	-2.470	8.882	-0.278	0.781		Denomination F3	-0.277	7.552	-0.037	0.971	
	Origin F1	-2.232	1.700	-1.313	0.190		Origin F1	0.883	1.447	0.610	0.542	
	Location F1	3.039	1.501	2.025	0.043	*	Location F1	-1.614	1.279	-1.262	0.207	
Remarried men	Intercept	20.997	1.159	18.114	< 2e-16	***	Intercept	13.039	0.900	14.486	< 2e-16	***
	Marriage age F	0.629	0.031	20.404	< 2e-16	***	Marriage age M	0.354	0.017	20.404	< 2e-16	***
	Occupation M2	-0.046	0.554	-0.083	0.934		Occupation M2	-0.957	0.415	-2.309	0.021	*
	Occupation M3	1.410	0.872	1.617	0.106		Occupation M3	-1.477	0.654	-2.259	0.024	*
	Occupation M4	3.452	0.837	4.123	0.000	***	Occupation M4	-2.304	0.629	-3.662	0.000	***
	Occupation M5	4.615	1.080	4.274	0.000	***	Occupation M5	-3.677	0.810	-4.542	0.000	***
	Occupation M6	2.573	1.026	2.507	0.012	*	Occupation M6	-1.424	0.771	-1.847	0.065	.
	Denomination M2	-0.114	1.089	-0.105	0.917		Denomination M2	0.825	0.817	1.011	0.312	
	Denomination M3	-4.028	4.833	-0.833	0.405		Denomination M3	4.497	3.625	1.241	0.215	
	Origin M1	0.278	0.729	0.381	0.703		Origin M1	0.234	0.547	0.428	0.668	
	Location M1	-0.961	0.749	-1.284	0.200		Location M1	1.477	0.561	2.635	0.009	**
	Denomination F2	-0.156	1.112	-0.141	0.888		Denomination F2	1.078	0.834	1.293	0.196	
	Denomination F3						Denomination F3					
	Origin F1	-0.645	0.959	-0.672	0.502		Origin F1	0.112	0.720	0.156	0.876	
	Location F1	0.920	0.797	1.154	0.249		Location F1	1.958	0.596	3.284	0.001	**
Remarried women	Intercept	20.523	1.069	19.207	< 2e-16	***	Intercept	20.744	1.324	15.663	< 2e-16	***
	Marriage age F	0.279	0.026	10.760	< 2e-16	***	Marriage age M	0.388	0.036	10.760	< 2e-16	***
	Occupation M2	-1.041	0.472	-2.205	0.028	*	Occupation M2	-0.740	0.558	-1.325	0.185	
	Occupation M3	0.312	0.593	0.526	0.599		Occupation M3	-0.438	0.700	-0.626	0.532	
	Occupation M4	4.231	0.868	4.877	0.000	***	Occupation M4	-0.960	1.036	-0.926	0.355	
	Occupation M5	0.886	0.884	1.003	0.316		Occupation M5	-0.541	1.043	-0.519	0.604	
	Occupation M6	-0.185	0.818	-0.226	0.821		Occupation M6	0.062	0.965	0.065	0.949	
	Denomination M2	0.016	0.922	0.017	0.986		Denomination M2	-1.763	1.087	-1.622	0.105	
	Denomination M3	2.330	3.290	0.708	0.479		Denomination M3	0.009	3.883	0.002	0.998	
	Origin M1	0.402	0.635	0.632	0.527		Origin M1	0.057	0.750	0.076	0.940	
	Location M1	-0.359	0.685	-0.523	0.601		Location M1	0.919	0.808	1.137	0.256	
	Denomination F2	-0.264	0.928	-0.285	0.776		Denomination F2	-0.362	1.095	-0.331	0.741	
	Denomination F3						Denomination F3					
	Origin F1	-1.417	0.798	-1.775	0.076	.	Origin F1	1.152	0.943	1.222	0.222	
	Location F1	1.912	0.678	2.819	0.005	**	Location F1	-0.061	0.803	-0.076	0.939	

Table S2 Analysis of Variance (ANOVA) for predictors of the age at marriage of men (Marriage age M) and women (Marriage age F) by marriage category. Significance codes: 0 '***' 0.001 '**' 0.01 '*' 0.05 '.' 0.1 ' ' 1

	Response: Marriage age M	Sum Sq	Df	F value	p value		Response: Marriage age F	Sum Sq	Df	F value	p value	
First marriages	Marriage age F	17014	1	852.254	< 2e-16	***	Marriage age M	19781	1	852.254	< 2e-16	***
	Occupation M	4859	5	48.678	< 2e-16	***	Occupation M	5218	5	44.959	< 2e-16	***
	Denomination M	9	2	0.228	0.796		Denomination M	71	2	1.525	0.218	
	Origin M	667	1	33.403	0.000	***	Origin M	647	1	27.883	0.000	***
	Location M	390	1	19.529	0.000	***	Location M	6	1	0.239	0.625	
	Denomination F	4	2	0.098	0.907		Denomination F	53	2	1.146	0.318	
	Origin F	17	1	0.845	0.358		Origin F	94	1	4.059	0.044	*
	Location F	171	1	8.549	0.003	**	Location F	2148	1	92.563	< 2e-16	***
Second marriages	Marriage age F	16288	1	207.857	< 2e-16	***	Marriage age M	11771.6	1	207.857	< 2e-16	***
	Occupation M	583	5	1.488	0.192		Occupation M	285.6	5	1.009	0.412	
	Denomination M	131	2	0.838	0.433		Denomination M	362.9	2	3.204	0.041	*
	Origin M	107	1	1.367	0.243		Origin M	15	1	0.266	0.606	
	Location M	45	1	0.568	0.451		Location M	16.5	1	0.291	0.590	
	Denomination F	92	2	0.590	0.555		Denomination F	167	2	1.475	0.230	
	Origin F	135	1	1.723	0.190		Origin F	21.1	1	0.372	0.542	
	Location F	321	1	4.102	0.043	*	Location F	90.3	1	1.594	0.207	
Remarried men	Marriage age F	28929	1	416.317	< 2e-16	***	Marriage age M	16284	1	416.317	< 2e-16	***
	Occupation M	2749	5	7.912	0.000	***	Occupation M	1110	5	5.675	0.000	***
	Denomination M	49	2	0.352	0.704		Denomination M	99	2	1.266	0.282	
	Origin M	10	1	0.145	0.703		Origin M	7	1	0.184	0.668	
	Location M	114	1	1.648	0.200		Location M	272	1	6.944	0.009	**
	Denomination F	1	1	0.020	0.888		Denomination F	65	1	1.673	0.196	
	Origin F	31	1	0.451	0.502		Origin F	1	1	0.024	0.876	
	Location F	93	1	1.333	0.249		Location F	422	1	10.784	0.001	**
Remarried women	Marriage age F	3691.3	1	115.773	< 2e-16	***	Marriage age M	5141	1	115.773	< 2e-16	***
	Occupation M	1286.4	5	8.069	0.000	***	Occupation M	116	5	0.521	0.760	
	Denomination M	16	2	0.251	0.778		Denomination M	117	2	1.316	0.269	
	Origin M	12.7	1	0.400	0.527		Origin M	0	1	0.006	0.940	
	Location M	8.7	1	0.274	0.601		Location M	57	1	1.293	0.256	
	Denomination F	2.6	1	0.081	0.776		Denomination F	5	1	0.109	0.741	
	Origin F	100.5	1	3.151	0.076	.	Origin F	66	1	1.494	0.222	
	Location F	253.4	1	7.949	0.005	**	Location F	0	1	0.006	0.939	
